# Validation of two automated ASPECTS software on non-contrast computed tomography scans of patients with acute ischemic stroke

**DOI:** 10.3389/fneur.2023.1170955

**Published:** 2023-04-06

**Authors:** Zhongping Chen, Zhenzhen Shi, Fei Lu, Linna Li, Mingyang Li, Shuo Wang, Wenxin Wang, Yongxin Li, Yu Luo, Dan Tong

**Affiliations:** ^1^Department of Radiology, The First Hospital of Jilin University, Changchun, China; ^2^Philips Healthcare, Beijing, China; ^3^Neusoft Medical Systems Co., Ltd., Shenyang, Liaoning, China; ^4^Department of Radiology, Shanghai Fourth People's Hospital, Shanghai, China

**Keywords:** image interpretation, computer-assisted, software validation, brain ischemia, computed tomography

## Abstract

**Purpose:**

The Alberta Stroke Program Early Computed Tomography Score (ASPECTS) was designed for semi-quantitative assessment of early ischemic changes on non-contrast computed tomography (NCCT) for acute ischemic stroke (AIS). We evaluated two automated ASPECTS software in comparison with reference standard.

**Methods:**

NCCT of 276 AIS patients were retrospectively reviewed (March 2018–June 2020). A three-radiologist consensus for ASPECTS was used as reference standard. Imaging data from both baseline and follow-up were evaluated for reference standard. Automated ASPECTS were calculated from baseline NCCT with 1-mm and 5-mm slice thickness, respectively. Agreement between automated ASPECTS and reference standard was assessed using intra-class correlation coefficient (ICC). Correlation of automated ASPECTS with baseline stroke severity (NIHSS) and follow-up ASPECTS were evaluated using Spearman correlation analysis.

**Results:**

In score-based analysis, automated ASPECTS calculated from 5-mm slice thickness images agreed well with reference standard (software A: ICC = 0.77; software B: ICC = 0.65). Bland–Altman analysis revealed that the mean differences between automated ASPECTS and reference standard were ≤ 0.6. In region-based analysis, automated ASPECTS derived from 5-mm slice thickness images by software A showed higher sensitivity (0.60 vs. 0.54), lower specificity (0.91 vs. 0.94), and higher AUC (0.76 vs. 0.74) than those using 1-mm slice thickness images (*p* < 0.05). Automated ASPECTS derived from 5-mm slice thickness images by software B showed higher sensitivity (0.56 vs. 0.51), higher specificity (0.87 vs. 0.81), higher accuracy (0.80 vs. 0.73), and higher AUC (0.71 vs. 0.66) than those using 1-mm slice thickness images (*p* < 0.05). Automated ASPECTS were significantly associated with baseline NIHSS and follow-up ASPECTS.

**Conclusion:**

Automated ASPECTS showed good reliability and 5 mm was the optimal slice thickness.

## Introduction

The Alberta Stroke Program Early Computed Tomography Score (ASPECTS) was introduced by PA Barber in 2000 and was designed for semi-quantitative assessment of early ischemic changes (EIC) on non-contrast computed tomography (NCCT) images of patients with acute ischemic stroke (AIS) (1). Since 2000, ASPECTS has been widely used to detect the extent of EIC, assess the effect of treatment, and to predict the prognosis (2). Guidelines for the early management of AIS recommend the use of ASPECTS to identify patients who are suited for mechanical thrombectomy (MT) (3). However, several factors may affect the assessment of ASPECTS (such as image acquisition, image quality, and doctor’s experience), and there is no clear consensus on the reliability of its use in the clinical workflow (2, 4).

Studies investigating the use of artificial intelligence technology for automated determination of ASPECTS have yielded robust results (5). Several automated software are available for calculation of ASPECTS using NCCT images (5). Studies using RAPID and e-ASPECTS software have yielded encouraging results (6). NBC software is now available at our hospital for automatic calculation of ASPECTS. In addition, there are available options for various parameters of NCCT images (e.g., slice thickness and reconstruction algorithms) (7, 8). Thinner slices and iterative reconstruction have been shown to improve the reliability and performance of automated ASPECTS (7–9). However, some studies have found no significant difference in ASPECTS automatically derived from images with different slice thicknesses (1 mm vs. 2.5 mm, ≤3 mm vs. 3–6 mm) (10, 11). Of note, in previous studies, not all the images with different slice thicknesses were reconstructed from raw data or the same patient (7, 10).

Validation studies of automated ASPECTS software also apply different reference standards for ASPECTS (7–11). Different reference standards have their inherent limitations. It is more scientific and reasonable to define the reference standard based on both baseline multimodal and follow-up images (6, 12).

The aim of this study was to assess the impact of different slice thicknesses of NCCT images on the performance of RAPID and NBC software, and to investigate the differences in the clinical utility of automated ASPECTS. Images with different slice thicknesses were reconstructed from the raw data of the same patient. A three-radiologist consensus for ASPECTS was used as the reference standard. Imaging data from both baseline and follow-up were evaluated for reference standard.

## Methods

The study protocol was approved by the Medical Ethics Committee of our hospital (Number 2020-380). The requirement for informed consent was waived off by the institutional review board owing to the retrospective nature of the analysis.

### Study design

**Figure 1:**
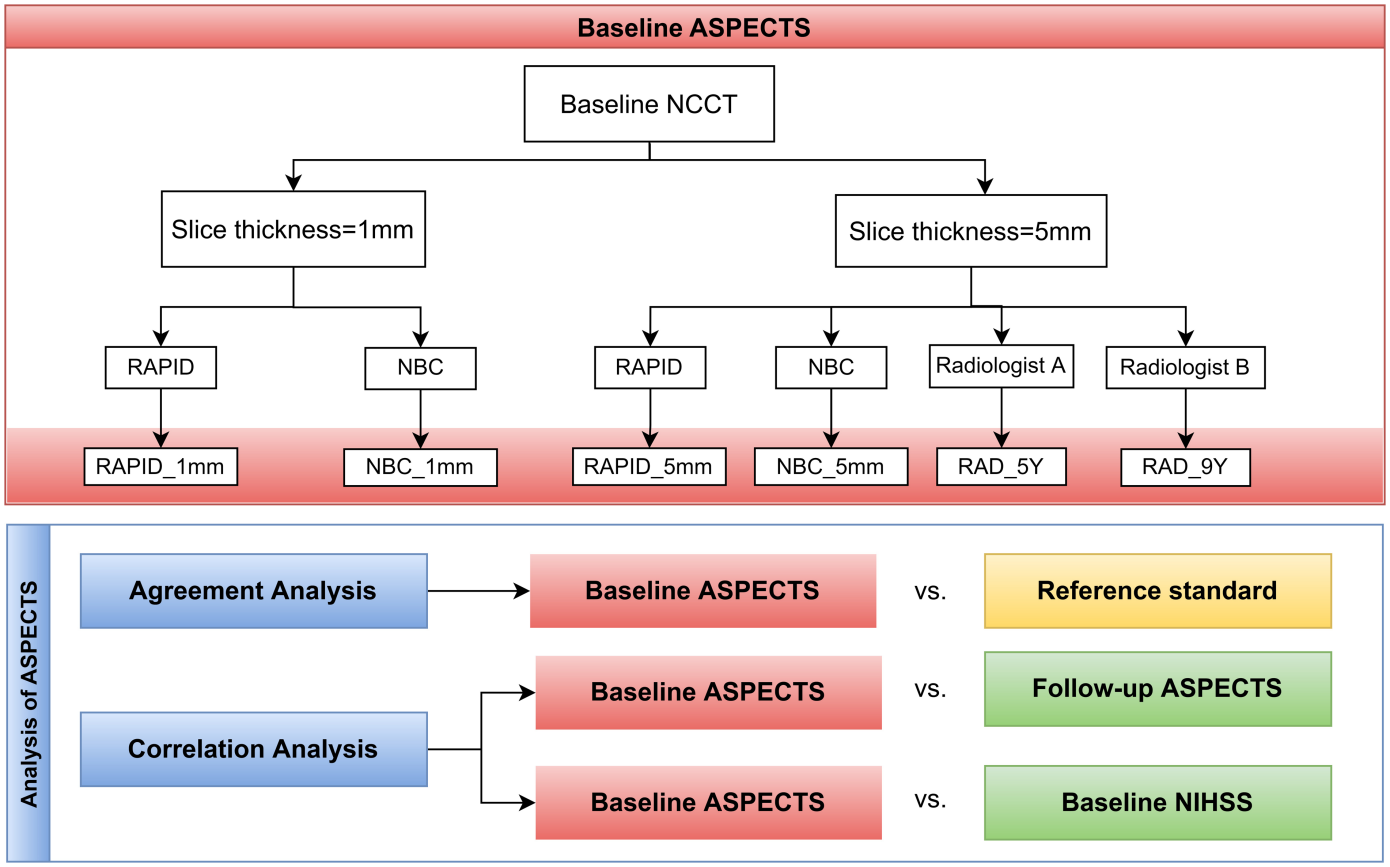
Workflow diagram of the study.

The present study was designed to evaluate the reliability and accuracy of automated ASPECTS based on images with slice thicknesses of 5 mm and 1 mm. Baseline ASPECTS were manually calculated from the baseline NCCT by two radiologists and automatically by the two software packages. Follow-up ASPECTS were generated by a single radiologist using follow-up CT/MRI. Reference standard of ASPECTS was defined by three radiologists. Imaging data from both baseline multimodal CT and follow-up CT/MRI were evaluated for the reference standard. Baseline ASPECTS were compared with the reference standard by means of score-based and region-based analysis. The correlation of baseline ASPECTS with baseline stroke severity (NIHSS) and follow-up ASPECTS were evaluated using Spearman correlation analysis. A schematic illustration of the study design is shown in Figure 1.

### Patient selection

**Figure 2:**
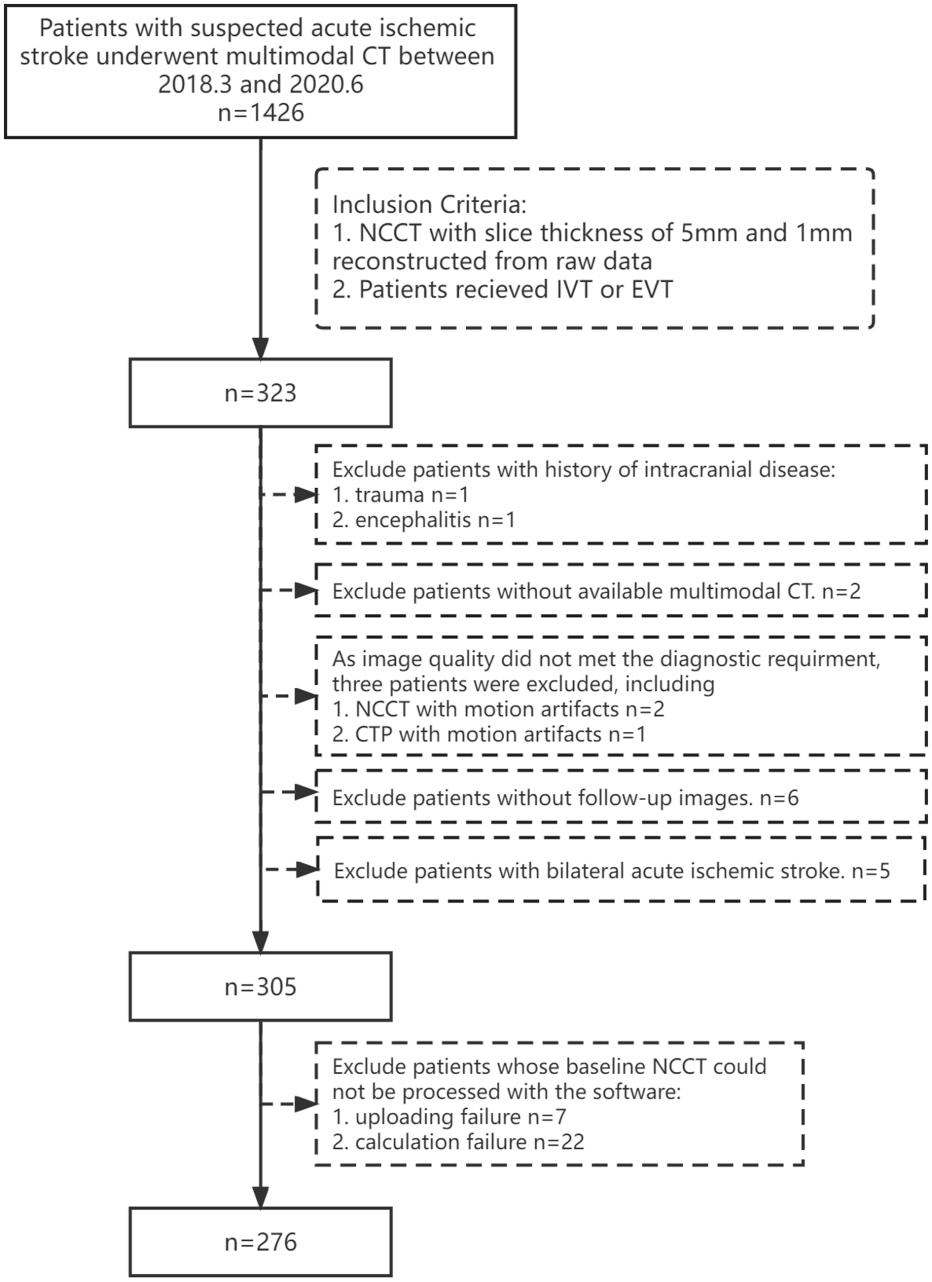
Flowchart showing the patient inclusion and exclusion criteria. IVT, intravenous thrombolysis; MT, mechanical thrombectomy.

We retrospectively enrolled patients with AIS who underwent multimodal CT scanning and received intravenous thrombolysis (IVT) or mechanical thrombectomy (MT) at the emergency department of our hospital between March 2018 and June 2020. Patients who qualified the following criteria were included: (1) age ≥ 18 years (2) availability of baseline multimodal CT images; (3) baseline NCCT images with 5 mm and 1 mm slice thickness reconstructed from raw data. The exclusion criteria were: (1) patients with hemorrhage, trauma, brain tumor, or encephalitis; (2) patients with bilateral AIS; (3) unqualified image quality for multimodal CT; (4) no follow-up CT/MRI obtained 24 h after baseline NCCT. The detailed flowchart of the patient selection process is shown in Figure 2.

### Image acquisition

Baseline multimodal CT imaging included brain NCCT, head and neck CT angiography (CTA), and brain CT perfusion (CTP). All baseline multimodal CT images were acquired using a 256-channel scanner (Brilliance 256; Philips Healthcare). Follow-up CT/MRI was obtained 24 h after the baseline NCCT. The scanning parameters for NCCT were: 120 kV, 200 mAs. The scanning parameters for CTP were 80 kV, 80 mAs, and 64 mm × 1.25 mm detector collimation and scan duration of 60 s. The scanning parameters for CTA were 100 kV, 266 mAs.

### Image analysis

#### Reference standard of ASPECTS

To define the reference standard, two radiologists (RAD_9Y and RAD_8Y, with 9 and 8 years of experience, respectively) independently reviewed the baseline multimodal CT and follow-up CT/MRI data. In the event of disagreement, a consensus score was provided by a third radiologist (RAD_30Y, with 30 years of experience). Meeting criteria 1 + 3 + 4 or 2 + 3 + 4 was defined as EIC on baseline NCCT: (1) presence of hypodensity and/or loss of gray-white differentiation on baseline NCCT (13), (2) relative mean transit time (rMTT) > 145%, cerebral blood volume (CBV) < 2.0 mL/100 g (14) or time-to-maximum (Tmax) > 6 s and relative cerebral blood flow (rCBF) < 30% on baseline CTP (15) (3) ischemic changes on follow-up CT/MRI; (4) Any individual region with EIC occupying ≥20% of that region was considered affected.

#### Manual ASPECTS

Three months later, the baseline NCCT images with slice thickness of 5 mm were retrospectively assessed by two radiologists (RAD_9Y and RAD_5Y, with 9 and 5 years of experience, respectively) without access to other imaging modality or clinical information, except for the lesion side. The ASPECTS calculated by the two radiologists were designated as RAD_9Y and RAD_5Y, respectively (as shown in Figure 1). There were no time restraints. Follow-up CT/MRI was retrospectively assessed by RAD_9Y. Modification of the window and level of NCCT was allowed as needed. Baseline NCCT images with slice thickness of 1 mm were allowed to be utilized by the radiologists.

#### Automated ASPECTS

The RAPID (iSchemaView, software version 5.0) and NBC (NeuBrainCARE) software supplied by the scanner’s manufacturers (Neusoft Medical Systems, software version 1.0) were used to automatically calculate ASPECTS. Both RAPID and NBC can analyze images with different slice thickness. Datasets with slice thicknesses of 2–3 mm and 5 mm are recommended for RAPID and NBC, respectively.

RAPID applies non-rigid registration with a standardized atlas to outline the 10 ASPECTS regions on each hemisphere. It measures the Hounsfield units (HU) and other parameters for the regions, then classifies each region as either normal or abnormal using a machine learning–based algorithm (random forest classifier) (6, 8). Finally, it calculates the ASPECTS.

NBC applies a non-rigid algorithm based on a hybrid supervised convolutional neural network (CNN) with a standardized atlas to outline the 10 ASPECTS regions on each hemisphere (16). It extracts the first order features, texture features and deep features. For the combined ASPECTS region, it recognizes the cerebral hemisphere with a lower CT value as the lesion side. Then, it classifies the ASPECTS regions as either normal or abnormal using a machine learning–based algorithm (weighted random forest classifier). Finally, it calculates the ASPECTS.

The ASPECTS automatically calculated using RAPID and NBC was designated as RAPID_5mm, RAPID_1mm, NBC_5mm, and NBC_1mm, according to the slice thickness (as shown in Figure 1).

### Statistical analysis

Continuous variables are presented as mean ± standard deviation (SD) or as median (interquartile range), while categorical data are presented as frequencies (percentage). In score-based analysis, Bland–Altman plots were used to illustrate differences between automated ASPECTS, manual ASPECTS, and the reference standard. The intra-class correlation coefficients (ICCs) were used to examine the agreement between automatic ASPECTS, manual ASPECTS, and the reference standard. Accuracy, sensitivity, specificity, and area under the curve (AUC) of the receiver-operating characteristics (ROC) curve were used to assess the performance of automated ASPECTS and manual ASPECTS methods compared to the reference standard at the region level. Correlation of the baseline and follow-up ASPECTS with the baseline neurological severity (NIHSS score) was estimated using Spearman correlation. Two-tailed *p*-value < 0.05 were considered indicative of statistical significance. Statistical analyses were performed using MedCalc (version 20.022).

## Results

### General information

A total of 276 eligible patients were retrospectively identified [median age: 64 years; interquartile range (IQR) 53–70 years; 71.01% male]. Baseline NCCT was obtained within a median time of 239 min from last known well (IQR 146–404 min); the median NIHSS score was 13 (IQR 9–16) and median consensus ASPECTS was 8.5 (IQR 6–10). The clinical and imaging characteristics are summarized in Table 1.

**Table 1:** Clinical and imaging characteristics of the study population.

Characteristics	Median (IQR) or *n* (%)
Sex	
Male	196 (71.01%)
Female	80 (28.99%)
Age	64 (53, 70)
Systolic blood pressure (mmHg)	150 (134, 169)
Diastolic blood pressure (mmHg)	87 (78, 98.5)
Glucose (mmol/L)	7.40 (6.30, 8.89)
Hemoglobin(g/L)	146 (135, 156)
Platelets (× 10^9^ cells per L)	202.50 (172, 241)
Baseline NIHSS	13 (9, 16)
Medical history	
Hypertension	158 (57.20%)
Previous stroke	54 (19.60%)
Coronary artery disease	38 (13.80%)
Atrial fibrillation	47 (17%)
Diabetes mellitus	51 (18.50%)
Hypercholesterolemia	17 (6.20%)
Current smoker	146 (52.90%)
Imaging features	
Symptom onset to NCCT (minutes)	239 (146, 404)
Difference between CT and CTP time (minutes)	3 (2, 5)
NCCT to treatment (minutes)	73 (53, 98)
Consensus ASPECTS	8.5 (6, 10)
C	66 (24%)
IC	47 (17%)
L	124 (45%)
I	97 (35%)
M1	38 (14%)
M2	70 (25%)
M3	35 (13%)
M4	41 (15%)
M5	101 (37%)
M6	45 (16%)
Follow-up ASPECTS	7 (4, 10)
Treatment	
Intravenous thrombolysis (IVT)	39
Mechanical thrombectomy (MT)	203
Bridging therapy (MT with prior IVT)	34

### ASPECTS score-level analysis

**Figure 3:**
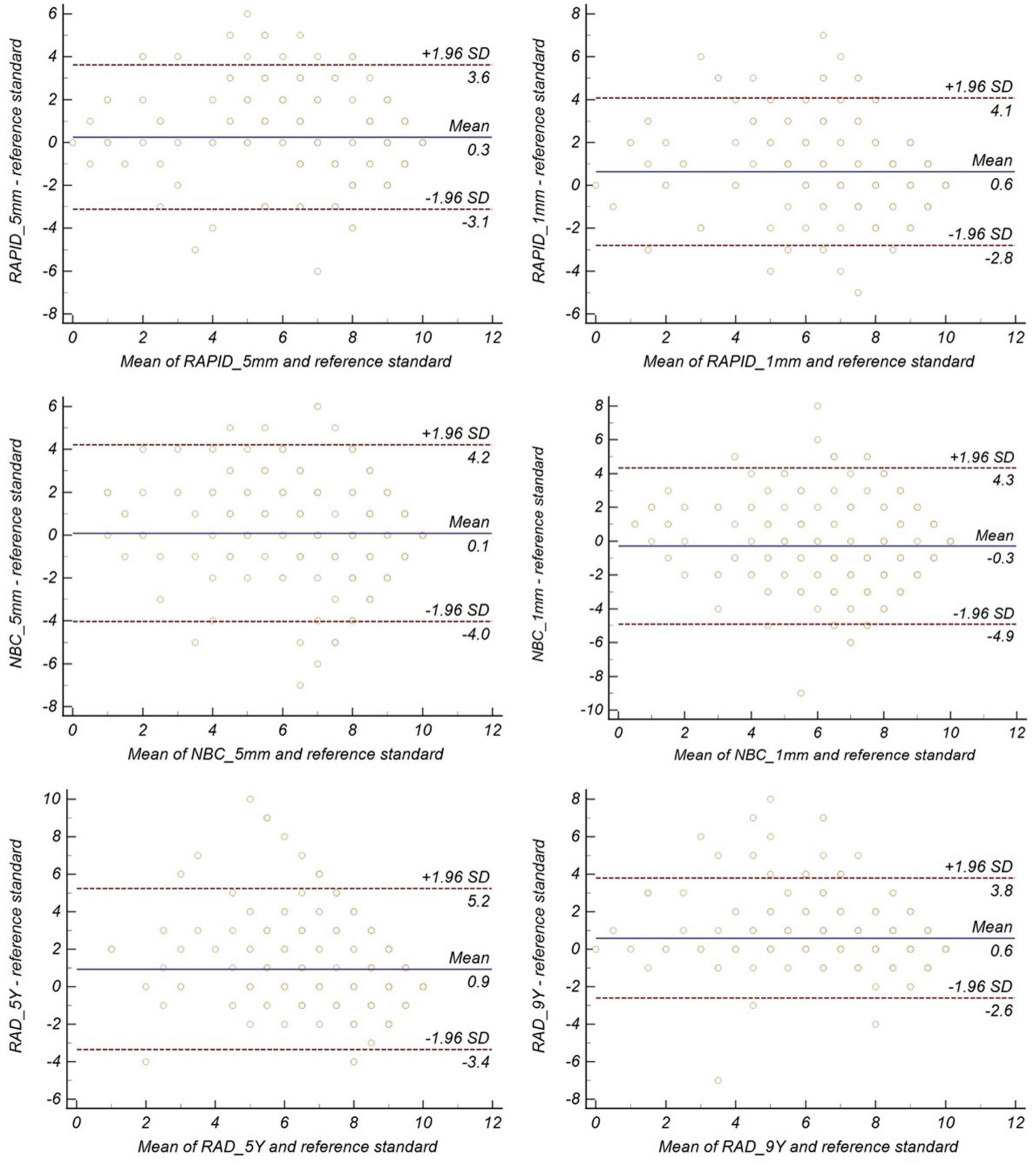
Bland–Altman plots for agreement between baseline ASPECTS and reference standard.

Bland–Altman analysis of the ASPECTS revealed that the mean differences between RAPID_5mm, RAPID_1mm, NBC_5mm, NBC_1mm, RAD_5Y, RAD_9Y, and reference standard were 0.3, 0.6, 0.1, −0.3, 0.9, and 0.6 points, respectively (Figure 3).

Automated ASPECTS derived from 5-mm slice thickness images showed higher agreement with reference standard than the scores derived from 1-mm slice thickness images (as shown in Table 2); however, the difference was not statistically significant.

**Table 2:** Agreement between baseline ASPECTS and reference standard.

	Slice thickness = 5 mm, ICC (95% CI)	Slice thickness = 1 mm, ICC (95% CI)
NBC	0.65 (0.58–0.72)	0.59 (0.51–0.66)
RAPID	0.77 (0.71–0.81)	0.74 (0.69–0.79)
RAD_5Y	0.57 (0.48–0.64)	
RAD_9Y	0.78 (0.73–0.82)	

ICC, Intra-class correlation coefficient; CI, Confidence interval.

### ASPECTS region-level analysis

**Figure 4:**
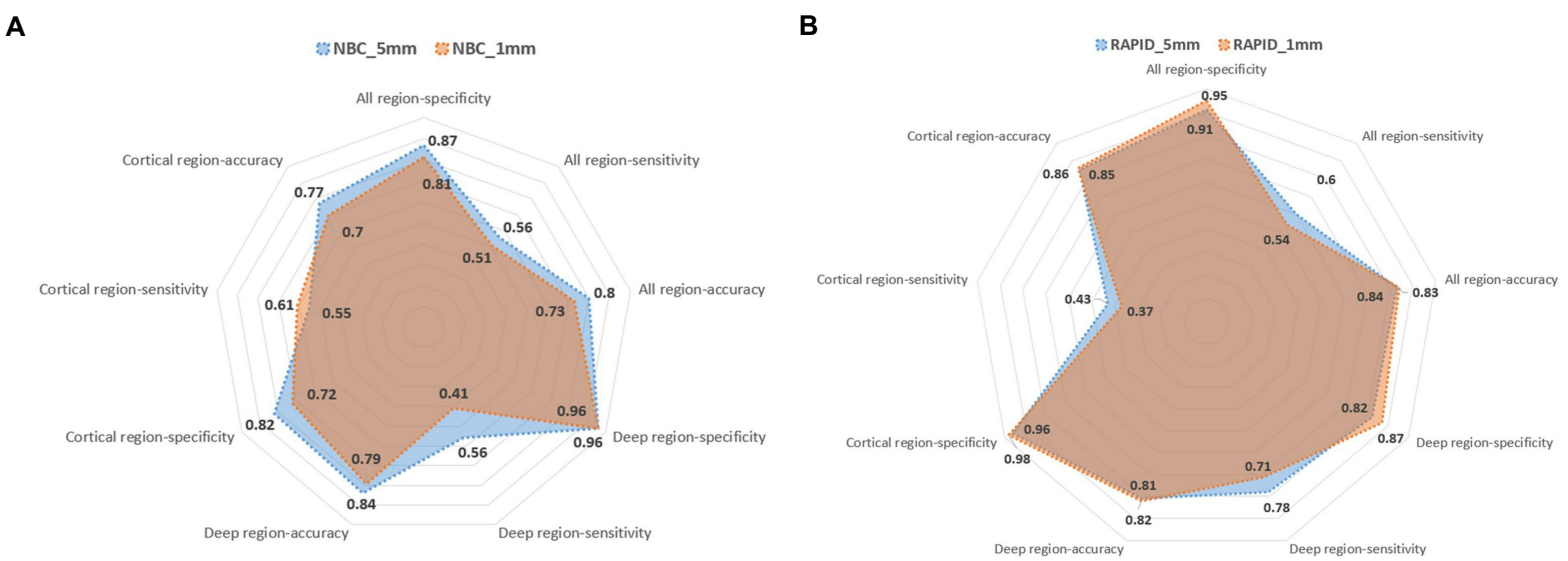
Visualization of the sensitivity, specificity and accuracy for automated ASPECTS calculated by NBC software **(A)** and RAPID software **(B)**.

For all ASPECTS regions combined, NBC_5mm showed better performance with higher sensitivity, specificity, accuracy, and AUC than NBC_1mm (*p* < 0.05), while RAPID_5mm showed better performance with higher sensitivity, lower specificity, and higher AUC than RAPID_1mm (*p* < 0.05; Table 3). Figure 4 shows a visualization of the sensitivity, specificity, and accuracy for automated ASPECTS derived from images with different slice thickness. Within the deep regions, NBC_5mm showed a tendency for higher sensitivity, greater accuracy, and similar specificity compared to NBC_1mm. RAPID_5mm showed a tendency for higher sensitivity, comparable accuracy, but lower specificity than RAPID_1mm. Within the cortical regions, NBC_5mm showed a tendency for higher specificity, but lower sensitivity and accuracy than NBC_1mm. RAPID_5mm showed a tendency for higher sensitivity, and similar accuracy and specificity compared to RAPID_1mm.

**Table 3:** Sensitivity, specificity, accuracy and AUCs of region-based analysis of ASPECTS.

Region	Rater	Slice thickness = 5mm				Slice thickness = 1mm			
		AUC	Accuracy	Specificity	Sensitivity	AUC	Accuracy	Specificity	Sensitivity
All region	NBC	0.71^#&^	0.80^#$&^	0.87^#$&^	0.56^#$&^	0.66^$&^	0.73^$&^	0.81^$&^	0.51^$&^
	RAPID	0.76^#$&^	0.83^&^	0.91^#$&^	0.60^#$&^	0.74^$&^	0.84^&^	0.94^&^	0.54^$&^
	RAD_5Y	0.70^&^	0.83^&^	0.95^&^	0.45^&^				
	RAD_9Y	0.82	0.9	0.98	0.66				
Deep Region	NBC	0.76^#$&^	0.84^#$&^	0.96	0.56^#$&^	0.68^&^	0.79^&^	0.96	0.41^&^
	RAPID	0.80^$&^	0.81^&^	0.82^#$&^	0.78^#$^	0.79^$&^	0.82^&^	0.87^$&^	0.71^$^
	RAD_5Y	0.71^&^	0.81^&^	0.96	0.46^&^				
	RAD_9Y	0.85	0.9	0.97	0.74				
Cortical Region	NBC	0.69^&^	0.77^#$&^	0.82^#$&^	0.55^#$^	0.66^&^	0.70^$&^	0.72^$&^	0.61^$^
	RAPID	0.69^&^	0.85^&^	0.96^#$&^	0.43^#&^	0.68^&^	0.86^&^	0.98^$^	0.37^&^
	RAD_5Y	0.69^&^	0.84^&^	0.94^&^	0.43^&^				
	RAD_9Y	0.79	0.9	0.98	0.59				

^#^indicates significant difference between the corresponding statistical indicators of ASPECTS derived from 5- and 1-mm slice thickness images. ^$^indicates significant difference between the corresponding statistical indicators of ASPECTS calculated by RAD_5Y and others. ^&^indicates significant difference between the corresponding statistical indicators of ASPECTS calculated by RAD_9Y and others.

**Figure 5:**
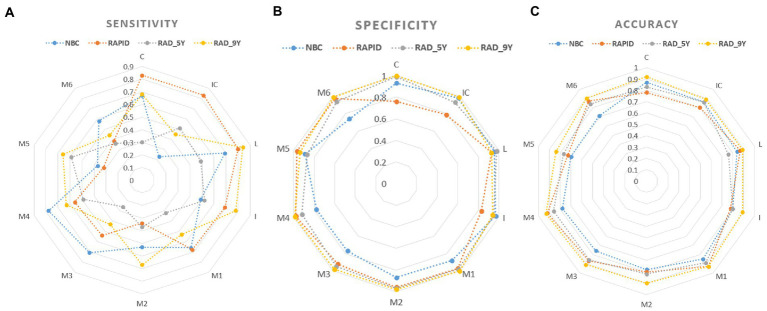
Visualization of the sensitivity **(A)**, specificity **(B)** and accuracy **(C)** for baseline ASPECTS.

Figure 5 shows the visualization of the sensitivity, specificity, and accuracy for baseline ASPECTS calculated by NBC, RAPID, RAD_5Y, and RAD_9Y based on 5-mm slice thickness images. The sensitivity, specificity, and accuracy of each ASPECTS region are shown in Supplementary Table 1. The AUCs and 95% CI of each ASPECTS region are shown in Supplementary Table 2. The *p-*values for comparison between two groups are shown in Supplementary Table 3.

### Correlation analysis

**Figure 6:**
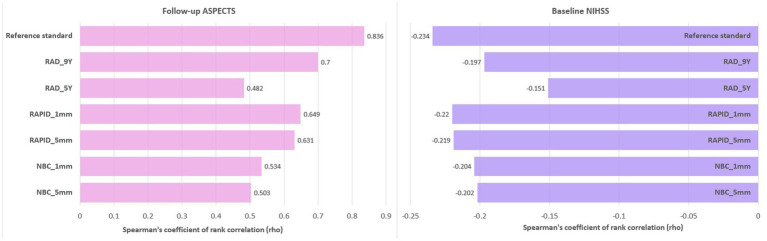
Bar plot of correlation coefficients (rho) between baseline NIHSS scores, follow-up ASPECTS, and baseline ASPECTS.

There was a significant correlation between baseline ASPECTS and follow-up ASPECTS as well as baseline stroke severity measured by NIHSS score (*p* ≤ 0.001). The detailed results for correlation coefficients (rho) are shown in Figure 6. The reference standard showed a strong correlation with follow-up ASPECTS (rho = 0.836). Decreasing baseline ASPECTS scores showed a significant correlation with increasing baseline NIHSS scores and decreasing follow-up ASPECTS scores.

## Discussion

In this study, automated ASPECTS produced robust scores for different slice thickness, especially with a stringent scanning protocol (120 kV, 200 mAs). Our study demonstrated that slice thickness had an influence on the automatic ASPECTS results. The ASPECTS automatically calculated from images with 5-mm slice thickness performed similar at score level and superior at region level to those calculated with 1-mm slice thickness.

Head NCCT is the most common examination for patients with AIS (17) and CT scanning protocol has a significant impact on ASPECTS. However, there is no stringent imaging protocol in different stroke centers, or even in the same hospital (18). Most radiomic features are highly affected by CT acquisition and reconstruction settings, especially in the intensity and texture categories (19). ASPECTS is a score based on subtle changes in density. Therefore, lack of a stringent imaging protocol for the assessment of ASPECTS introduces greater variability. In this study, we applied a stringent imaging protocol in the Philips 256 section scanner. Compared with previous automated ASPECTS software validation studies (120–140 kV, 300–320 mAs) (20, 21), we applied lower tube voltage and tube current (120 kV, 200 mAs). In the study by Fletcher et al., the performance of lower-dose head CT with 200 mAs was non-inferior to that of the standard protocol for detection of intracranial findings (22). However, there is a lack of evidence demonstrating the effect of scanning parameters (120 kV, 200 mAs) on the performance of automatic ASPECTS software. Our study demonstrates that automated ASPECTS software produced reliable and robust results with the scanning parameters used (120 kV, 200 mAs).

There is no gold standard for ASPECTS assessment (6) and the rigor of reference standard directly affects the accuracy of ASPECTS evaluation. The reference standard used for assessment of the reliability of automatic ASPECTS varied in previous studies (11, 12, 23). The conclusions may vary due to the different reference standard applied. Even experienced doctors show only a fair degree of agreement (kappa = 0.56–0.57) in ASPECTS evaluation (6). Due to subtle changes in AIS, using consensus score obtained by experts from the baseline NCCT as the reference standard is liable to overestimate the ASPECTS score (8). Due to the time delay between the baseline NCCT and reperfusion, ASPECTS obtained using follow-up images as the reference standard is liable to be underestimated (11). Due to successful recanalization, diffusion weighted imaging (DWI) or CTP lesions can be reversed; thus ASPECTS based on baseline DWI/CTP as the reference standard would be underestimated (21, 24). In order to define the most accurate reference standard for ASPECTS, it is advisable to utilize baseline NCCT, CTP, and follow-up images (6, 12). This is in line with the present study. In the present study, the median time delay between baseline NCCT and CTP was 3 min (IQR 2, 5), which is much lower than the time delay (1 or 2 h) between baseline NCCT and DWI applied in other studies (25, 26). The definition of reference standards is one of the strengths of the present study.

Slice thickness had an influence on automatic ASPECTS results, however, the conclusions of different studies are inconsistent. Compared to slice thickness of 5 mm, RAPID ASPECTS were optimal at 0.9 mm slice thickness (kappa = 0.777 vs. 0.814) (8). RAPID ASPECTS derived from 2.5 mm slice thickness showed higher kappa than those from slice thickness of 1.0 mm images (kappa = 0.24 vs. 0.19) (11). Our study demonstrates that NBC and RAPID calculations using 5-mm slice thickness images performed better than those using images with 1-mm slice thickness at score level (*p* > 0.05) and region level (*p* < 0.05). The reason for different conclusions may be the scanning protocol (current study: 120 kV, 200 mAs; Austein’s study (11): 120 kV, 250 mAs; Loffler’s study (8): 120 kV, 300 mAs). Another reason may be reconstruction algorithm (current study: hybrid iterative reconstruction, level 3; Austein’s study (11): hybrid iterative reconstruction, level 2; Loffler’s study (8): iterative model reconstruction). Reconstruction algorithm can improve the image quality of brain NCCT and improve the accuracy of ASPECTS. However, the better the reconstruction algorithm, the longer the calculation time, and the higher the requirements on the hardware. Additionally, the standard slice thickness generally used for brain NCCT is 5 mm (27). There is a beneficial reduction in image noise for thicker slices. Therefore, application of 5 mm slice thickness is more conducive to the application and promotion of automated ASPECTS, especially in lower-level stroke centers. 5 mm slice thickness should be the optimal choice.

ASPECTS is highly correlated with baseline neurological severity in AIS. e-ASPECTS derived from 1-mm slice thickness images showed the highest correlation with the baseline NIHSS score (7). Of note, the additional axial images with slice thickness of 2–10 mm were generated from 1-mm slice thickness reconstructions, and not raw data (7). In a previous study, ASPECTS derived from ≤6-mm slice thickness showed a strong correlation with baseline NIHSS score; however, the images with two different slice thicknesses compared were from different patients (10). To minimize additional sources of variability, different slice thicknesses images in the current study were derived from the raw data and the same patient. However, only a subtle increase in the correlation coefficients was noted for slice thicknesses of 1 mm compared to 5 mm. Automated ASPECTS scores from images with 1- and 5-mm slice thickness were highly correlated with baseline NIHSS score and follow-up ASPECTS.

Accurate segmentation of ASPECTS region is the basis for region specific accuracy of automated ASPECTS. At region level, automated ASPECTS show greater differences on sensitivity than specificity and accuracy. NBC showed higher specificity and lower sensitivity, while RAPID showed lower specificity and higher sensitivity for detection of EIC in deep regions. NBC showed lower specificity and higher sensitivity, while RAPID showed higher specificity and lower sensitivity for detection of EIC in cortical regions. NBC and RAPID did not perform better than experienced radiologists for detection of EIC in cortical regions. Variability in region level results may be due to inconsistent definition of the region boundaries, especially for IC and M4-6 (11). The volume of IC is small and its boundary is obscure. The cortical regions used by NBC only include cortical tissue; however, RAPID includes part of the periventricular white matter. Both software apply standardized atlas method to outline ASPECTS regions in each hemisphere. Application of standardized atlas is technically challenging owing to stringent imaging protocols (28). Brain atrophy and atypical brains affect the segmentation accuracy (28, 29). Therefore, the accuracy of ASPECTS region segmentation is crucial, and applying a standardized atlas may not be the best option. Future studies are required to explore ASPECTS segmentation accuracy.

Some limitations of our study should be considered. Firstly, we used imaging data from a single center. NCCT image acquisition and quality tend to vary in different centers. However, the sample size of this study was relatively large. Secondly, this study did not analyze the correlation between automated ASPECTS and prognosis. Several factors are known to affect the functional outcomes of AIS, such as onset time, NIHSS score, age, occlusion site, and treatment protocol. We included patients who received IVT or MT; hence we did not assess the prognostic value of ASPECTS. However, we analyzed the correlation between baseline NIHSS score, follow-up ASPECTS, and baseline ASPECTS.

## Conclusion

Automated ASPECTS produced robust scores with a stringent scanning protocol (120 kV, 200 mAs). Our results indicate that slice thickness impacts the identification of EIC by automated ASPECTS, and that the optimal slice thickness is 5 mm. Different software offer different advantages for assessment of deep regions and cortical regions.

## Data availability statement

The original contributions presented in the study are included in the article/Supplementary material, further inquiries can be directed to the corresponding author.

## Ethics statement

The studies involving human participants were reviewed and approved by the Medical Ethics Committee of the First Hospital of Jilin University Clinical trial and research approval. Written informed consent for participation was not required for this study in accordance with the national legislation and the institutional requirements.

## Author contributions

DT and ZC: full access to all of the data in the study and takes responsibility for the integrity of the data and the accuracy of the data analysis. ZS, FL, and LL: data collection. DT and ZC: study concept and design. ZC: drafting of the manuscript. DT: critical revision of the manuscript for important intellectual content. ML and SW: statistical analysis. ZC: study supervision. DT, ZC, and WW: CT scanning protocol. WW, YLi, and YLuo: supplying software. All authors contributed to the article and approved the submitted version.

## Funding

This study has received funding by the Jilin Province Special Scientific Research Project of Health (2018SCZWSZX-001), with additional funding support from the Jilin Province Science and Technology Development Plan Project (20200201303JC).

## Conflict of interest

YLi was an employee of Neusoft Medical Systems Co., Ltd. WW was an employee of Philips Healthcare.

The remaining authors declare that the research was conducted in the absence of any commercial or financial relationships that could be construed as a potential conflict of interest.

## Publisher’s note

All claims expressed in this article are solely those of the authors and do not necessarily represent those of their affiliated organizations, or those of the publisher, the editors and the reviewers. Any product that may be evaluated in this article, or claim that may be made by its manufacturer, is not guaranteed or endorsed by the publisher.

## Abbreviations

ASPECTS: Alberta Stroke Program Early Computed Tomography Score
EIC: Early ischemic changes
NCCT: Non-contrast computed tomography
AIS: Acute ischemic stroke
MCA: Middle cerebral artery
IVT: Intravenous thrombolysis
MT: Mechanical thrombectomy
CNR: Contrast-to-noise-ratio
CTP: CT perfusion
HU: Hounsfield units
IQR: Interquartile range
ICC: Intra-class correlation coefficient
AUC: Area under the curve
ROC: Receiver-operating characteristics
