# Supplementary material for: Validation of two automated ASPECTS software on non-contrast computed tomography scans of patients with acute ischemic stroke
Source: Front Neurol. 2023 Apr 6;14:1170955. doi: 10.3389/fneur.2023.1170955 (PMC10116051; doi:10.3389/fneur.2023.1170955)
Supplement: Supplementary file 1 [file Table_1.DOCX]

**Supplementary Table 1 Specificity, sensitivity and accuracy for each region**

|  |  | **5-mm, Acc / Spe / Sen** | **1-mm, Acc / Spe / Sen** |
| --- | --- | --- | --- |
| **C** | NBC | 0.87**^&^** /0.93**^$&^** /0.67**^$^** | 0.83**^&^** /0.92**^$&^**/0.55**^$^** |
|  | RAPID | 0.78**^&^** /0.76**^$&^** /0.83**^$&^** | 0.80**^&^** /0.81**^$&^** /0.76**^$^** |
|  | RAD_5Y | 0.83**^&^** /0.99 /0.30**^&^** |  |
|  | RAD_9Y | 0.92 /1.00 /0.68 |  |
| **IC** | NBC | 0.86**^&^** /0.98**^$^** /0.23**^$&^** | 0.82**^&^** /0.96 /0.15**^$&^** |
|  | RAPID | 0.80**^#$&^** /0.79**^#$&^** /0.83**^$&^** | 0.84 /0.86**^$&^** /0.77**^$&^** |
|  | RAD_5Y | 0.86 /0.93**^&^** /0.51 |  |
|  | RAD_9Y | 0.89 /0.99 /0.45 |  |
| **L** | NBC | 0.84**^#$^** /0.96 /0.69**^#$&^** | 0.74**^&^** /0.98 /0.45**^&^** |
|  | RAPID | 0.87**^$^** /0.93**^$^** /0.80**^#$^** | 0.84**^$^** /0.93 /0.72**^$&^** |
|  | RAD_5Y | 0.76**^&^** /0.98 /0.49**^&^** |  |
|  | RAD_9Y | 0.89 /0.93 /0.84 |  |
| **I** | NBC | 0.80**^&^** /0.97 /0.49**^#&^** | 0.78**^&^** /0.99**^&^** /0.38**^$&^** |
|  | RAPID | 0.78**^&^** /0.83**^#$&^** /0.69**^$^** | 0.80**^&^** /0.90 /0.63**^&^** |
|  | RAD_5Y | 0.80**^&^** /0.95 /0.52**^&^** |  |
|  | RAD_9Y | 0.89 /0.94 /0.78 |  |
| **M1** | NBC | 0.85**^#&^** /0.88**^#$&^** /0.66**^$^** | 0.79**^$&^** /0.80**^$&^** /0.68**^$^** |
|  | RAPID | 0.93 /0.97**^&^** /0.68**^$^** | 0.92 /0.98 /0.55**^$^** |
|  | RAD_5Y | 0.89**^&^** /0.98 /0.32 |  |
|  | RAD_9Y | 0.93 /1.00 /0.53 |  |
| **M2** | NBC | 0.78**^#&^** /0.87**^#$&^** /0.53**^$^** | 0.69**^$&^** /0.69**^$&^** /0.67**^$^** |
|  | RAPID | 0.80**^&^** /0.96 /0.34**^&^** | 0.82**^&^** /0.98 /0.34**^&^** |
|  | RAD_5Y | 0.82**^&^** /0.97 /0.37**^&^** |  |
|  | RAD_9Y | 0.90 /0.98 /0.67 |  |
| **M3** | NBC | 0.76**^#$&^** /0.77**^#$&^** /0.71**^$&^** | 0.66**^$&^** /0.65**^$&^** /0.69**^$&^** |
|  | RAPID | 0.87 /0.92**^#&^** /0.54 | 0.89 /0.96 /0.40 |
|  | RAD_5Y | 0.86**^&^** /0.95 /0.26 |  |
|  | RAD_9Y | 0.91 /0.98 /0.43 |  |
| **M4** | NBC | 0.78**^$&^** /0.78**^$&^** /0.78**^$^** | 0.72**^$&^** /0.72**^$&^** /0.73**^$^** |
|  | RAPID | 0.92**^$^** /0.98**^$^** /0.56 | 0.91**^$^** /0.99**^$^** /0.49 |
|  | RAD_5Y | 0.86**^&^** /0.92**^&^** /0.49 |  |
|  | RAD_9Y | 0.93 /0.99 /0.63 |  |
| **M5** | NBC | 0.70**^$&^** /0.89 /0.37**^$&^** | 0.70**^&^** /0.86**^&^** /0.42**^$&^** |
|  | RAPID | 0.73**^&^** /0.97**^$^** /0.32**^$&^** | 0.73**^&^** /0.98**^$&^** /0.30**^$&^** |
|  | RAD_5Y | 0.77**^&^** /0.87**^&^** /0.59 |  |
|  | RAD_9Y | 0.84 /0.94 /0.66 |  |
| **M6** | NBC | 0.71**^#$&^** /0.74**^#$&^** /0.58 | 0.62**^$&^** /0.60**^$&^** /0.73**^$&^** |
|  | RAPID | 0.87 /0.97 /0.38 | 0.87 /0.97 /0.31 |
|  | RAD_5Y | 0.84**^&^** /0.94**^&^** /0.36 |  |
|  | RAD_9Y | 0.90 /0.99 /0.44 |  |

**NOTE: ACC** = accuracy, **Spe** = specificity, **Sen** = sensitivity.

**^#^** indicates significant difference between the corresponding statistical indicators of ASPECTS derived from 5-mm and 1-mm slice thickness images;

**^$^** indicates significant difference between the corresponding statistical indicators of ASPECTS calculated by RAD_5Y and others;

**^&^** indicates significant difference between the corresponding statistical indicators of ASPECTS calculated by RAD_9Y and others.
