# Supplementary material for: Validation of two automated ASPECTS software on non-contrast computed tomography scans of patients with acute ischemic stroke
Source: Front Neurol. 2023 Apr 6;14:1170955. doi: 10.3389/fneur.2023.1170955 (PMC10116051; doi:10.3389/fneur.2023.1170955)
Supplement: Supplementary file 2 [file Table_2.DOCX]

**Supplementary Table 2 AUC and 95% Confidence Intervals (CI) for each region**

|  |  | **5-mm, AUC (95% CI)** | **1-mm, AUC (95% CI)** |
| --- | --- | --- | --- |
| **C** | NBC | 0.80 (0.75, 0.84)**^$^** | 0.73 (0.68, 0.78)**^$&^** |
|  | RAPID | 0.80 (0.75, 0.84)**^$^** | 0.78 (0.73, 0.83)**^$^** |
|  | RAD_5Y | 0.65 (0.59, 0.70)**^&^** |  |
|  | RAD_9Y | 0.84 (0.79, 0.88) |  |
| **IC** | NBC | 0.61 (0.55, 0.67)**^$&^** | 0.56 (0.49, 0.61)**^$&^** |
|  | RAPID | 0.81 (0.76, 0.86)**^$&^** | 0.81 (0.76, 0.86)**^$&^** |
|  | RAD_5Y | 0.72 (0.67, 0.78) |  |
|  | RAD_9Y | 0.72 (0.66, 0.77) |  |
| **L** | NBC | 0.82 (0.77, 0.87)**^$&#^** | 0.72 (0.66, 0.77)**^&^** |
|  | RAPID | 0.86 (0.82, 0.90)**^$^** | 0.83 (0.78, 0.87)**^$&^** |
|  | RAD_5Y | 0.74 (0.68, 0.79)**^&^** |  |
|  | RAD_9Y | 0.88 (0.84, 0.92) |  |
| **I** | NBC | 0.73 (0.68, 0.79)**^&#^** | 0.69 (0.63, 0.74)**^&^** |
|  | RAPID | 0.76 (0.70, 0.81)**^&^** | 0.76 (0.71, 0.81)**^&^** |
|  | RAD_5Y | 0.73 (0.68, 0.78)**^&^** |  |
|  | RAD_9Y | 0.86 (0.82, 0.90) |  |
| **M1** | NBC | 0.77 (0.72, 0.82)**^$^** | 0.74 (0.69, 0.79) |
|  | RAPID | 0.83 (0.78, 0.87)**^$#^** | 0.77 (0.71, 0.81)**^$^** |
|  | RAD_5Y | 0.65 (0.59, 0.71)**^&^** |  |
|  | RAD_9Y | 0.76 (0.71, 0.81) |  |
| **M2** | NBC | 0.70 (0.64, 0.75)**^&^** | 0.68 (0.62, 0.74)**^&^** |
|  | RAPID | 0.65 (0.59, 0.71)**^&^** | 0.66 (0.60, 0.72)**^&^** |
|  | RAD_5Y | 0.67 (0.61, 0.72)**^&^** |  |
|  | RAD_9Y | 0.83 (0.78, 0.87) |  |
| **M3** | NBC | 0.74 (0.69, 0.79)**^$^** | 0.67 (0.61, 0.72) |
|  | RAPID | 0.73 (0.68, 0.78)**^$^** | 0.68 (0.62, 0.74) |
|  | RAD_5Y | 0.60 (0.54, 0.66)**^&^** |  |
|  | RAD_9Y | 0.71 (0.65, 0.76) |  |
| **M4** | NBC | 0.78 (0.73, 0.83) | 0.73 (0.67, 0.78) |
|  | RAPID | 0.77 (0.72, 0.82) | 0.74 (0.68, 0.79) |
|  | RAD_5Y | 0.71 (0.65, 0.76)**^&^** |  |
|  | RAD_9Y | 0.81 (0.76, 0.86) |  |
| **M5** | NBC | 0.63 (0.57, 0.69)**^$&^** | 0.64 (0.58, 0.69)**^$&^** |
|  | RAPID | 0.64 (0.58, 0.70)**^$&^** | 0.64 (0.58, 0.70)**^$&^** |
|  | RAD_5Y | 0.73 (0.68, 0.78)**^&^** |  |
|  | RAD_9Y | 0.80 (0.75, 0.85) |  |
| **M6** | NBC | 0.66 (0.60, 0.72) | 0.67 (0.61, 0.72) |
|  | RAPID | 0.67 (0.61, 0.73) | 0.64 (0.58, 0.70) |
|  | RAD_5Y | 0.65 (0.59, 0.70) |  |
|  | RAD_9Y | 0.72 (0.66, 0.77) |  |

**NOTE:**

**^#^** indicates significant difference between the corresponding statistical indicators of ASPECTS derived from 5-mm and 1-mm slice thickness images;

**^$^** indicates significant difference between the corresponding statistical indicators of ASPECTS calculated by RAD_5Y and others;

**^&^** indicates significant difference between the corresponding statistical indicators of ASPECTS calculated by RAD_9Y and others.

.
