# Supplementary material for: Validation of two automated ASPECTS software on non-contrast computed tomography scans of patients with acute ischemic stroke
Source: Front Neurol. 2023 Apr 6;14:1170955. doi: 10.3389/fneur.2023.1170955 (PMC10116051; doi:10.3389/fneur.2023.1170955)
Supplement: Supplementary file 3 [file Table_3.DOCX]

**Supplementary Table 3 The P value for comparison between two groups**

| Comparison | Sensitivity | Specificity | Accuracy | AUC |
| --- | --- | --- | --- | --- |
| All region |  |  |  |  |
| NBC_1mm vs. NBC_5mm | 0.0152 | < 0.0001 | < 0.0001 | < 0.0001 |
| NBC_1mm vs. RAPID_1mm | 0.2031 | < 0.0001 | < 0.0001 | < 0.0001 |
| NBC_1mm vs. RAPID_5mm | 0.0001 | < 0.0001 | < 0.0001 | < 0.0001 |
| NBC_1mm vs. RAD_5Y | 0.0281 | < 0.0001 | < 0.0001 | 0.0034 |
| NBC_1mm vs. RAD_9Y | < 0.0001 | < 0.0001 | < 0.0001 | < 0.0001 |
| NBC_5mm vs. RAPID_1mm | 0.4871 | < 0.0001 | < 0.0001 | 0.0313 |
| NBC_5mm vs. RAPID_5mm | 0.0332 | 0.0001 | < 0.0001 | 0.0003 |
| NBC_5mm vs. RAD_5Y | < 0.0001 | < 0.0001 | 0.0003 | 0.2529 |
| NBC_5mm vs. RAD_9Y | < 0.0001 | < 0.0001 | < 0.0001 | < 0.0001 |
| RAPID_1mm vs. RAPID_5mm | < 0.0001 | < 0.0001 | 0.1910 | 0.0444 |
| RAPID_1mm vs. RAD_5Y | 0.0004 | 0.1224 | 0.0952 | 0.0021 |
| RAPID_1mm vs. RAD_9Y | < 0.0001 | < 0.0001 | < 0.0001 | < 0.0001 |
| RAPID_5mm vs. RAD_5Y | < 0.0001 | < 0.0001 | 0.5194 | < 0.0001 |
| RAPID_5mm vs. RAD_9Y | 0.0096 | < 0.0001 | < 0.0001 | < 0.0001 |
| RAD_5Y vs. RAD_9Y | < 0.0001 | < 0.0001 | < 0.0001 | < 0.0001 |
| Deep region |  |  |  |  |
| NBC_1mm vs. NBC_5mm | < 0.0001 | 1.0000 | < 0.0001 | < 0.0001 |
| NBC_1mm vs. RAPID_1mm | < 0.0001 | < 0.0001 | 0.0511 | < 0.0001 |
| NBC_1mm vs. RAPID_5mm | < 0.0001 | < 0.0001 | 0.4119 | < 0.0001 |
| NBC_1mm vs. RAD_5Y | 0.1098 | 0.8957 | 0.1412 | 0.0899 |
| NBC_1mm vs. RAD_9Y | < 0.0001 | 0.4966 | < 0.0001 | < 0.0001 |
| NBC_5mm vs. RAPID_1mm | < 0.0001 | < 0.0001 | 0.1471 | 0.1066 |
| NBC_5mm vs. RAPID_5mm | < 0.0001 | < 0.0001 | 0.0154 | 0.0207 |
| NBC_5mm vs. RAD_5Y | 0.0057 | 1.0000 | 0.0250 | 0.0064 |
| NBC_5mm vs. RAD_9Y | < 0.0001 | 0.5327 | < 0.0001 | < 0.0001 |
| RAPID_1mm vs. RAPID_5mm | 0.0043 | 0.0003 | 0.2661 | 0.4732 |
| RAPID_1mm vs. RAD_5Y | < 0.0001 | < 0.0001 | 0.5564 | < 0.0001 |
| RAPID_1mm vs. RAD_9Y | 0.3729 | < 0.0001 | < 0.0001 | 0.0001 |
| RAPID_5mm vs. RAD_5Y | < 0.0001 | < 0.0001 | 0.7627 | < 0.0001 |
| RAPID_5mm vs. RAD_9Y | 0.1750 | < 0.0001 | < 0.0001 | 0.0008 |
| RAD_5Y vs. RAD_9Y | < 0.0001 | 0.6718 | < 0.0001 | < 0.0001 |
| Cortical region |  |  |  |  |
| NBC_1mm vs. NBC_5mm | 0.0330 | < 0.0001 | < 0.0001 | 0.1546 |
| NBC_1mm vs. RAPID_1mm | < 0.0001 | < 0.0001 | < 0.0001 | 0.4817 |
| NBC_1mm vs. RAPID_5mm | < 0.0001 | < 0.0001 | < 0.0001 | 0.0591 |
| NBC_1mm vs. RAD_5Y | < 0.0001 | < 0.0001 | < 0.0001 | 0.2762 |
| NBC_1mm vs. RAD_9Y | 0.6208 | < 0.0001 | < 0.0001 | < 0.0001 |
| NBC_5mm vs. RAPID_1mm | < 0.0001 | < 0.0001 | < 0.0001 | 0.5029 |
| NBC_5mm vs. RAPID_5mm | < 0.0001 | < 0.0001 | < 0.0001 | 0.6021 |
| NBC_5mm vs. RAD_5Y | 0.0023 | < 0.0001 | < 0.0001 | 0.9193 |
| NBC_5mm vs. RAD_9Y | 0.3156 | < 0.0001 | < 0.0001 | < 0.0001 |
| RAPID_1mm vs. RAPID_5mm | 0.0051 | 0.0001 | 0.5666 | 0.0555 |
| RAPID_1mm vs. RAD_5Y | 0.1279 | < 0.0001 | 0.0926 | 0.5262 |
| RAPID_1mm vs. RAD_9Y | < 0.0001 | 0.5424 | < 0.0001 | < 0.0001 |
| RAPID_5mm vs. RAD_5Y | 0.9354 | 0.0323 | 0.1995 | 0.7532 |
| RAPID_5mm vs. RAD_9Y | < 0.0001 | 0.0003 | < 0.0001 | < 0.0001 |
| RAD_5Y vs. RAD_9Y | < 0.0001 | < 0.0001 | < 0.0001 | < 0.0001 |
| C |  |  |  |  |
| NBC_1mm vs. NBC_5mm | 0.0963 | 0.8450 | 0.1742 | 0.0529 |
| NBC_1mm vs. RAPID_1mm | 0.0066 | 0.0003 | 0.3135 | 0.1754 |
| NBC_1mm vs. RAPID_5mm | 0.0003 | < 0.0001 | 0.1507 | 0.0956 |
| NBC_1mm vs. RAD_5Y | 0.0025 | 0.0003 | 1.0000 | 0.0214 |
| NBC_1mm vs. RAD_9Y | 0.0931 | 0.0001 | 0.0001 | 0.0040 |
| NBC_5mm vs. RAPID_1mm | 0.3075 | 0.0001 | 0.0248 | 0.7237 |
| NBC_5mm vs. RAPID_5mm | 0.0266 | < 0.0001 | 0.0043 | 1.0000 |
| NBC_5mm vs. RAD_5Y | < 0.0001 | 0.0023 | 0.1263 | < 0.0001 |
| NBC_5mm vs. RAD_9Y | 1.0000 | 0.0001 | 0.0135 | 0.2338 |
| RAPID_1mm vs. RAPID_5mm | 0.3323 | 0.1934 | 0.6201 | 0.6894 |
| RAPID_1mm vs. RAD_5Y | < 0.0001 | < 0.0001 | 0.4160 | 0.0002 |
| RAPID_1mm vs. RAD_9Y | 0.3018 | < 0.0001 | < 0.0001 | 0.0872 |
| RAPID_5mm vs. RAD_5Y | < 0.0001 | < 0.0001 | 0.2031 | 0.0001 |
| RAPID_5mm vs. RAD_9Y | 0.0414 | < 0.0001 | < 0.0001 | 0.2549 |
| RAD_5Y vs. RAD_9Y | < 0.0001 | 1.0000 | < 0.0001 | < 0.0001 |
| IC |  |  |  |  |
| NBC_1mm vs. NBC_5mm | 0.2891 | 0.2266 | 0.0636 | 0.0808 |
| NBC_1mm vs. RAPID_1mm | < 0.0001 | 0.0002 | 0.5446 | < 0.0001 |
| NBC_1mm vs. RAPID_5mm | < 0.0001 | < 0.0001 | 0.4999 | < 0.0001 |
| NBC_1mm vs. RAD_5Y | 0.0002 | 0.3075 | 0.1352 | 0.0001 |
| NBC_1mm vs. RAD_9Y | 0.0026 | 0.1460 | 0.0005 | 0.0002 |
| NBC_5mm vs. RAPID_1mm | < 0.0001 | < 0.0001 | 0.7914 | < 0.0001 |
| NBC_5mm vs. RAPID_5mm | < 0.0001 | < 0.0001 | 0.0846 | < 0.0001 |
| NBC_5mm vs. RAD_5Y | 0.0072 | 0.0127 | 0.8714 | 0.0125 |
| NBC_5mm vs. RAD_9Y | 0.0414 | 1.0000 | 0.0433 | 0.0176 |
| RAPID_1mm vs. RAPID_5mm | 0.4531 | 0.0052 | 0.0470 | 0.9209 |
| RAPID_1mm vs. RAD_5Y | 0.0042 | 0.0023 | 0.5515 | 0.0249 |
| RAPID_1mm vs. RAD_9Y | 0.0026 | < 0.0001 | 0.0814 | 0.0423 |
| RAPID_5mm vs. RAD_5Y | 0.0003 | < 0.0001 | 0.0247 | 0.0280 |
| RAPID_5mm vs. RAD_9Y | 0.0001 | < 0.0001 | 0.0016 | 0.0338 |
| RAD_5Y vs. RAD_9Y | 0.5488 | 0.0042 | 0.1221 | 0.8752 |
| L |  |  |  |  |
| NBC_1mm vs. NBC_5mm | < 0.0001 | 0.3750 | 0.0001 | < 0.0001 |
| NBC_1mm vs. RAPID_1mm | < 0.0001 | 0.0391 | 0.0005 | < 0.0001 |
| NBC_1mm vs. RAPID_5mm | < 0.0001 | 0.0215 | < 0.0001 | < 0.0001 |
| NBC_1mm vs. RAD_5Y | 0.5515 | 1.0000 | 0.5758 | 0.4759 |
| NBC_1mm vs. RAD_9Y | < 0.0001 | 0.0574 | < 0.0001 | < 0.0001 |
| NBC_5mm vs. RAPID_1mm | 0.5716 | 0.4240 | 1.0000 | 0.9041 |
| NBC_5mm vs. RAPID_5mm | 0.0094 | 0.2668 | 0.1996 | 0.0852 |
| NBC_5mm vs. RAD_5Y | 0.0007 | 0.5078 | 0.0075 | 0.0022 |
| NBC_5mm vs. RAD_9Y | 0.0019 | 0.3018 | 0.0649 | 0.0218 |
| RAPID_1mm vs. RAPID_5mm | 0.0309 | 1.0000 | 0.1360 | 0.0646 |
| RAPID_1mm vs. RAD_5Y | < 0.0001 | 0.0654 | 0.0038 | 0.0004 |
| RAPID_1mm vs. RAD_9Y | 0.0107 | 1.0000 | 0.0595 | 0.0264 |
| RAPID_5mm vs. RAD_5Y | < 0.0001 | 0.0386 | 0.0001 | < 0.0001 |
| RAPID_5mm vs. RAD_9Y | 0.4049 | 1.0000 | 0.5327 | 0.3983 |
| RAD_5Y vs. RAD_9Y | < 0.0001 | 0.0574 | < 0.0001 | < 0.0001 |
| I |  |  |  |  |
| NBC_1mm vs. NBC_5mm | 0.0266 | 0.3750 | 0.1686 | 0.0428 |
| NBC_1mm vs. RAPID_1mm | 0.0001 | < 0.0001 | 0.3317 | 0.0092 |
| NBC_1mm vs. RAPID_5mm | < 0.0001 | < 0.0001 | 1.0000 | 0.0243 |
| NBC_1mm vs. RAD_5Y | 0.0410 | 0.0654 | 0.4614 | 0.1289 |
| NBC_1mm vs. RAD_9Y | < 0.0001 | 0.0215 | < 0.0001 | < 0.0001 |
| NBC_5mm vs. RAPID_1mm | 0.0294 | 0.0010 | 1.0000 | 0.3051 |
| NBC_5mm vs. RAPID_5mm | 0.0003 | < 0.0001 | 0.4101 | 0.3706 |
| NBC_5mm vs. RAD_5Y | 0.8746 | 0.4240 | 0.8919 | 0.9800 |
| NBC_5mm vs. RAD_9Y | < 0.0001 | 0.1250 | 0.0008 | < 0.0001 |
| RAPID_1mm vs. RAPID_5mm | 0.3075 | 0.0192 | 0.4011 | 0.8525 |
| RAPID_1mm vs. RAD_5Y | 0.1352 | 0.0784 | 0.9022 | 0.3878 |
| RAPID_1mm vs. RAD_9Y | 0.0135 | 0.0963 | 0.0018 | 0.0013 |
| RAPID_5mm vs. RAD_5Y | 0.0076 | 0.0002 | 0.6353 | 0.4419 |
| RAPID_5mm vs. RAD_9Y | 0.1221 | 0.0001 | 0.0001 | 0.0005 |
| RAD_5Y vs. RAD_9Y | < 0.0001 | 1.0000 | 0.0005 | < 0.0001 |
| M1 |  |  |  |  |
| NBC_1mm vs. NBC_5mm | 1.0000 | 0.0066 | 0.0198 | 0.5269 |
| NBC_1mm vs. RAPID_1mm | 0.2266 | < 0.0001 | < 0.0001 | 0.6150 |
| NBC_1mm vs. RAPID_5mm | 1.0000 | < 0.0001 | < 0.0001 | 0.0396 |
| NBC_1mm vs. RAD_5Y | 0.0094 | < 0.0001 | 0.0013 | 0.1327 |
| NBC_1mm vs. RAD_9Y | 0.2379 | < 0.0001 | < 0.0001 | 0.7547 |
| NBC_5mm vs. RAPID_1mm | 0.3877 | < 0.0001 | 0.0034 | 0.9262 |
| NBC_5mm vs. RAPID_5mm | 1.0000 | 0.0002 | 0.0011 | 0.2250 |
| NBC_5mm vs. RAD_5Y | 0.0044 | < 0.0001 | 0.1608 | 0.0207 |
| NBC_5mm vs. RAD_9Y | 0.3323 | < 0.0001 | 0.0013 | 0.8688 |
| RAPID_1mm vs. RAPID_5mm | 0.0625 | 0.3750 | 0.7539 | 0.0348 |
| RAPID_1mm vs. RAD_5Y | 0.0490 | 1.0000 | 0.1686 | 0.0247 |
| RAPID_1mm vs. RAD_9Y | 1.0000 | 0.2188 | 0.6476 | 0.9216 |
| RAPID_5mm vs. RAD_5Y | 0.0026 | 0.3877 | 0.1102 | 0.0007 |
| RAPID_5mm vs. RAD_9Y | 0.2101 | 0.0391 | 1.0000 | 0.2177 |
| RAD_5Y vs. RAD_9Y | 0.0768 | 0.3750 | 0.0266 | 0.0277 |
| M2 |  |  |  |  |
| NBC_1mm vs. NBC_5mm | 0.0755 | < 0.0001 | 0.0067 | 0.6891 |
| NBC_1mm vs. RAPID_1mm | < 0.0001 | < 0.0001 | 0.0002 | 0.5830 |
| NBC_1mm vs. RAPID_5mm | < 0.0001 | < 0.0001 | 0.0010 | 0.4228 |
| NBC_1mm vs. RAD_5Y | 0.0015 | < 0.0001 | 0.0007 | 0.7572 |
| NBC_1mm vs. RAD_9Y | 1.0000 | < 0.0001 | < 0.0001 | 0.0018 |
| NBC_5mm vs. RAPID_1mm | 0.0106 | < 0.0001 | 0.2026 | 0.2871 |
| NBC_5mm vs. RAPID_5mm | 0.0106 | 0.0003 | 0.4799 | 0.1799 |
| NBC_5mm vs. RAD_5Y | 0.0433 | 0.0002 | 0.2717 | 0.4153 |
| NBC_5mm vs. RAD_9Y | 0.1102 | < 0.0001 | < 0.0001 | 0.0021 |
| RAPID_1mm vs. RAPID_5mm | 1.0000 | 0.1250 | 0.3877 | 0.6425 |
| RAPID_1mm vs. RAD_5Y | 0.8601 | 0.5488 | 1.0000 | 0.8658 |
| RAPID_1mm vs. RAD_9Y | < 0.0001 | 1.0000 | 0.0001 | < 0.0001 |
| RAPID_5mm vs. RAD_5Y | 0.8506 | 1.0000 | 0.7552 | 0.6685 |
| RAPID_5mm vs. RAD_9Y | < 0.0001 | 0.1250 | < 0.0001 | < 0.0001 |
| RAD_5Y vs. RAD_9Y | 0.0001 | 0.5078 | 0.0001 | < 0.0001 |
| M3 |  |  |  |  |
| NBC_1mm vs. NBC_5mm | 1.0000 | 0.0001 | 0.0002 | 0.1041 |
| NBC_1mm vs. RAPID_1mm | 0.0063 | < 0.0001 | < 0.0001 | 0.7840 |
| NBC_1mm vs. RAPID_5mm | 0.1250 | < 0.0001 | < 0.0001 | 0.1093 |
| NBC_1mm vs. RAD_5Y | 0.0015 | < 0.0001 | < 0.0001 | 0.2632 |
| NBC_1mm vs. RAD_9Y | 0.0225 | < 0.0001 | < 0.0001 | 0.4538 |
| NBC_5mm vs. RAPID_1mm | 0.0034 | < 0.0001 | < 0.0001 | 0.1866 |
| NBC_5mm vs. RAPID_5mm | 0.1796 | < 0.0001 | 0.0002 | 0.8383 |
| NBC_5mm vs. RAD_5Y | 0.0001 | < 0.0001 | 0.0032 | 0.0051 |
| NBC_5mm vs. RAD_9Y | 0.0309 | < 0.0001 | < 0.0001 | 0.5240 |
| RAPID_1mm vs. RAPID_5mm | 0.1250 | 0.0129 | 0.3833 | 0.1724 |
| RAPID_1mm vs. RAD_5Y | 0.3593 | 0.6291 | 0.2430 | 0.2146 |
| RAPID_1mm vs. RAD_9Y | 1.0000 | 0.1797 | 0.2863 | 0.6390 |
| RAPID_5mm vs. RAD_5Y | 0.0525 | 0.2295 | 0.7709 | 0.0458 |
| RAPID_5mm vs. RAD_9Y | 0.4240 | 0.0007 | 0.0801 | 0.6303 |
| RAD_5Y vs. RAD_9Y | 0.1460 | 0.0386 | 0.0066 | 0.0350 |
| M4 |  |  |  |  |
| NBC_1mm vs. NBC_5mm | 0.6875 | 0.0980 | 0.0681 | 0.1144 |
| NBC_1mm vs. RAPID_1mm | 0.0129 | < 0.0001 | < 0.0001 | 0.8228 |
| NBC_1mm vs. RAPID_5mm | 0.0654 | < 0.0001 | < 0.0001 | 0.3073 |
| NBC_1mm vs. RAD_5Y | 0.0414 | < 0.0001 | 0.0001 | 0.6865 |
| NBC_1mm vs. RAD_9Y | 0.4545 | < 0.0001 | < 0.0001 | 0.1022 |
| NBC_5mm vs. RAPID_1mm | 0.0018 | < 0.0001 | < 0.0001 | 0.2961 |
| NBC_5mm vs. RAPID_5mm | 0.0225 | < 0.0001 | < 0.0001 | 0.7838 |
| NBC_5mm vs. RAD_5Y | 0.0118 | < 0.0001 | 0.0238 | 0.1474 |
| NBC_5mm vs. RAD_9Y | 0.1796 | < 0.0001 | < 0.0001 | 0.5363 |
| RAPID_1mm vs. RAPID_5mm | 0.2500 | 0.5000 | 1.0000 | 0.1202 |
| RAPID_1mm vs. RAD_5Y | 1.0000 | 0.0015 | 0.0201 | 0.5258 |
| RAPID_1mm vs. RAD_9Y | 0.1460 | 1.0000 | 0.2379 | 0.0779 |
| RAPID_5mm vs. RAD_5Y | 0.6072 | 0.0106 | 0.0139 | 0.1855 |
| RAPID_5mm vs. RAD_9Y | 0.6072 | 0.7266 | 0.4049 | 0.3934 |
| RAD_5Y vs. RAD_9Y | 0.1460 | 0.0007 | 0.0002 | 0.0127 |
| M5 |  |  |  |  |
| NBC_1mm vs. NBC_5mm | 0.3593 | 0.3075 | 1.0000 | 0.7670 |
| NBC_1mm vs. RAPID_1mm | 0.0227 | < 0.0001 | 0.2026 | 0.8997 |
| NBC_1mm vs. RAPID_5mm | 0.0639 | 0.0003 | 0.2624 | 0.8636 |
| NBC_1mm vs. RAD_5Y | 0.0247 | 0.8746 | 0.0544 | 0.0209 |
| NBC_1mm vs. RAD_9Y | 0.0002 | 0.0201 | < 0.0001 | < 0.0001 |
| NBC_5mm vs. RAPID_1mm | 0.2100 | 0.0001 | 0.2110 | 0.6744 |
| NBC_5mm vs. RAPID_5mm | 0.3833 | 0.0044 | 0.2682 | 0.6300 |
| NBC_5mm vs. RAD_5Y | 0.0014 | 0.5966 | 0.0448 | 0.0052 |
| NBC_5mm vs. RAD_9Y | < 0.0001 | 0.1516 | < 0.0001 | < 0.0001 |
| RAPID_1mm vs. RAPID_5mm | 0.7266 | 0.4531 | 1.0000 | 0.9335 |
| RAPID_1mm vs. RAD_5Y | 0.0001 | < 0.0001 | 0.3082 | 0.0111 |
| RAPID_1mm vs. RAD_9Y | < 0.0001 | 0.0386 | 0.0002 | < 0.0001 |
| RAPID_5mm vs. RAD_5Y | 0.0001 | 0.0002 | 0.2416 | 0.0110 |
| RAPID_5mm vs. RAD_9Y | < 0.0001 | 0.2668 | 0.0001 | < 0.0001 |
| RAD_5Y vs. RAD_9Y | 0.3105 | 0.0227 | 0.0183 | 0.0329 |
| M6 |  |  |  |  |
| NBC_1mm vs. NBC_5mm | 0.0654 | 0.0002 | 0.0073 | 0.8735 |
| NBC_1mm vs. RAPID_1mm | < 0.0001 | < 0.0001 | < 0.0001 | 0.5768 |
| NBC_1mm vs. RAPID_5mm | 0.0001 | < 0.0001 | < 0.0001 | 0.8851 |
| NBC_1mm vs. RAD_5Y | 0.0005 | < 0.0001 | < 0.0001 | 0.7157 |
| NBC_1mm vs. RAD_9Y | 0.0044 | < 0.0001 | < 0.0001 | 0.2611 |
| NBC_5mm vs. RAPID_1mm | 0.0018 | < 0.0001 | < 0.0001 | 0.6796 |
| NBC_5mm vs. RAPID_5mm | 0.0225 | < 0.0001 | < 0.0001 | 0.7553 |
| NBC_5mm vs. RAD_5Y | 0.0755 | < 0.0001 | 0.0003 | 0.8402 |
| NBC_5mm vs. RAD_9Y | 0.2632 | < 0.0001 | < 0.0001 | 0.2517 |
| RAPID_1mm vs. RAPID_5mm | 0.4531 | 0.6875 | 1.0000 | 0.3300 |
| RAPID_1mm vs. RAD_5Y | 0.8145 | 0.1153 | 0.4177 | 0.9195 |
| RAPID_1mm vs. RAD_9Y | 0.2101 | 0.2188 | 0.0525 | 0.0878 |
| RAPID_5mm vs. RAD_5Y | 1.0000 | 0.2863 | 0.3240 | 0.5894 |
| RAPID_5mm vs. RAD_9Y | 0.6476 | 0.0703 | 0.1221 | 0.3455 |
| RAD_5Y vs. RAD_9Y | 0.3877 | 0.0042 | 0.0037 | 0.0730 |
